# Superelasticity Evaluation of the Biocompatible Ti-17Nb-6Ta Alloy

**DOI:** 10.1155/2019/8353409

**Published:** 2019-01-08

**Authors:** Alaa Keshtta, Mohamed A.-H. Gepreel

**Affiliations:** Department of Materials Science and Engineering, Egypt-Japan University of Science and Technology, New Borg El-Arab, Alexandria 21934, Egypt

## Abstract

Recently, studying the shape memory effect of the biocompatible Ti alloys takes much attention in the biomedical and healthcare applications. This study concerns about characterizing the superelasticity of the new biocompatible Ti-17Nb-6Ta (TNT) alloy. Microstructure of TNT was observed using optical and confocal microscopes. The alloy consists of two phases: *β* (predominant phase) and *α*″ martensite phase. The influence of cold rolling deformation on the microstructure was illustrated in which the martensitic-induced transformation appeared by cold rolling. The alloy is ductile as only the fracture dimples appeared in its fracture surface. Multicyclic loading and deloading tensile testing was applied to TNT specimens (flat and wire shapes) in order to evaluate the superelasticity. A superelastic strain as high as 3.5% was recorded for this TNT alloy. Therefore, TNT alloy has high potential for many biomedical and healthcare applications.

## 1. Introduction

Recently, shape memory alloys (SMAs) covered great areas in all biomedical, healthcare, and industrial fields as they become one of the main partners in most precise applications. Also, SMAs are known as the multifunctional material or active material. Active materials have a mechanical response although it is not exposed to mechanical field [1]. SMAs are widely spread in the last decades as it is involved in many industries such as aerospace, automotive, and biomedical applications [2, 3]. The unique properties of the SMA open the opportunities for complicated applications as the material response may generate a motion or apply a force due to the shape memory effect. It can also restore energy of the deformation which results from the superelastic effect [1, 4, 5].

In the last two decades, the shape memory alloys became a big interest for the researcher, a candidate in biomedical applications. The increasing demand for new materials due to the expanding applications in biomedical fields resulted in more patents which exceeded half of the shape memory alloys patents in US over a 13-year-period from 1990 to 2013, as the patents in the biomedical field counted about 61% of the total SMA patents [2]. The SMAs are already contributing in enormous biomedical applications such as orthodontics (braces, palatals arches, and files), orthopedics (bone, spine, head, muscles, hand, and legs), and vascular system (aorta, arteries, valves, vena cava filter, ventricular septal defect, and vessels) [6–10].

In the last decades, Nitinol had overrun many industries such as aerospace, automotive mini actuators, micro-electromechanical systems, and biomedical applications [2]. In the biomedical branch, Nitinol is used widely in orthodontic wires and stents. Superelasticity is the secret behind the attraction to Nitinol which proved to have an elastic recovery up to 8% that equals 16 times of 316 stainless steel. This interesting mechanical behavior of Nitinol resulted in high demands in the different marketing fields, especially in the medical ones [6]. The unique properties of Nitinol include less corrosion tendency and higher biocompatibility, leading to dramatic changes in biomaterial usage by replacing stainless steels alloys. On the contrary, a noticeable disadvantage was the higher cost of this alloy. In short, Nitinol became the most presenting SMAs in the biomedical field for decades [2, 6].

However, unfortunately Ni was proved to cause allergy for some patient [11, 12], and this made it necessary to search for a new material that relatively has higher biocompatibility. Also, nickel was proved to be a toxic element [11]; in result, looking for alternatives became a must to overcome the side effects of Ni in Nitinol. New researches focused on achieving the required biocompatibility as long as mechanical properties by using nickel-free SMAs [13–24].

This alloy was investigated by Gepreel [25]. The alloy showed good corrosion resistance compared with Ti-6Al-4V as it exceeded more than ten times [26]. Also the mechanical properties were investigated in the previous studies which proved good workability and ductility, besides achieving controllable strength [23, 26]. Also, a new study concerned to investigate the effect of adding the zirconium element for the TNT alloy on both microstructure and mechanical properties [27]. The potential of the TNT alloy to work as drug-eluting stents is also studied recently and showed very promising results [28].

The aim of this work is to investigate the new biocompatible Ti-17Nb-6Ta (TNT) alloy as a new candidate shape memory alloy. This Ni-free proposed shape memory alloy is designed to be used in biomedical stents and orthodontic application instead of the most commonly used alloys (i.e., Nitinol) which proved to cause allergy to a number of patients. The superelastic properties of the new TNT alloy had not been fully characterized before, so this study is proposed to catch the unknown trends of it with a focus on the superelasticity.

## 2. Experimental Procedures

### 2.1. Material Preparation

**Figure 1:**
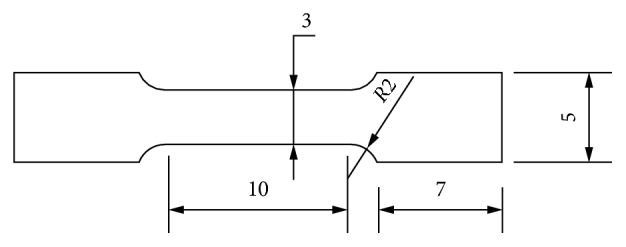
Specimen dimension of the flat cold-rolled plate. All dimensions are in mm.

This alloy was designed to be in the *β*/(*β* + *α*″) phase boundary region at which elastic softening appeared in this *β-*phase alloy [29, 30]. High-purity alloying elements, titanium, niobium, and tantalum, were used to produce the Ti-17Nb-6Ta cast alloy by an electric arc furnace (ARCAST 200, USA) in argon atmosphere. The alloy was turned upside down (flipped) and remelted twice in the furnace to get the ultimate homogeneity. After that, solution treatment was carried at 900°C for 30 min in argon atmosphere and then quenched in water. Then, the specimens were divided into two groups to obtain cold-rolled samples. The first group flat cold-rolled deformed specimens with a reduction ratio 95% in the thickness in which the specimens were cut into the dimensions illustrated in Figure 1 using the water jet cutting machine. The second group samples were subjected to square cold rolling deformation. The wire had square cross section area with dimension 1.3 ∗ 1.3 mm^2^ and length of 60 mm.

Solution treatment was applied on some specimens in the tube furnace at 900°C for 30 min in argon atmosphere followed by ice-water quenching. By using different sand papers grades, oxide layers were removed. Microstructure analysis was applied in order to investigate microstructural changes of the alloys resulted from cold rolling deformation. The optical micrographs were observed using the optical microscope and confocal microscope with different magnifications. The chemical etching solution with concentrations of 8% HF (40% conc.) and 8% HNO_3_ (70% conc.) for 20 seconds was used. For all specimens groups, the loading and deloading cyclic tensile test was applied using the universal testing machine (Shimadzu AGS-X, 100 kN, with a crosshead speed of 3.33 × 10^−6^ m·s^−1^ in air at room temperature) up to a maximum of 600 MPa and 650 MPa engineering tensile stress. The fracture surfaces of fractured tensile test samples were investigated using the laser microscope, and the 2D surface and 3D images were plotted.

## 3. Results and Discussion

**Figure 2:**
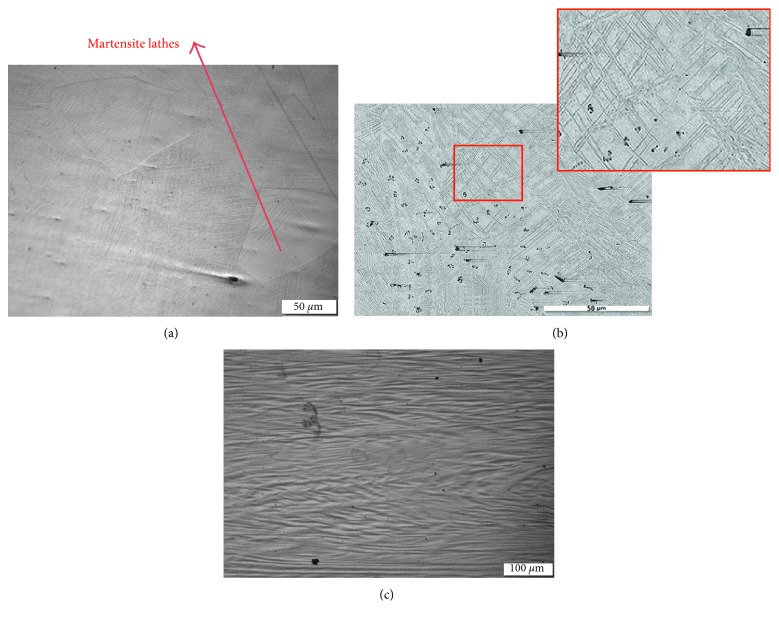
Optical microstructure of the (a) solution-treated wire, (b) cold-rolled wire with 30% reduction in the cross-sectional area, and (c) cold-rolled wire with 90% reduction in the cross-sectional area.

To study the microstructures change with deformation and heat treatment, the effects of various deformation reduction ratios on the microstructure of the TNT alloy were observed, as shown in Figure 2. In Figure 2, images of solution-treated wire and cold-rolled wires with reduction ratios of 30% and 90% are studied. In general, the martensite phase appeared predominantly in all conditions.

The microstructure of the solution-treated wire is shown in Figure 2(a). It can be seen in Figure 2(a) that the grains are generally equiaxed and the grain sizes are around 150 *μ*m. Also, the martensite lathes clearly appear inside the grains and at the grain boundaries. After 30% reduction was carried on wire specimens by cold rolling in the cross-sectional area, the influence of deformation is clearly appeared as shown in Figure 2(b). It was found that the martensite lathes seem increased compared to the ones found in the solution-treated wire. Another observation to mention is that the orientation of martensite lathes is much more random and the lathes were intersectionally crossed in the same grain. This means that there are some induced changes by deformation in the martensite phase in terms of the amount and orientation. Again, these microstructural optical images showed different microstructures of the martensite phase as the volume fraction increased, and the morphology seems different too after cold rolling by 30% reduction in the cross-sectional area. The changes in the martensite phases after deformation not only are proved by the microstructure images but also appeared in the XRD result as reported in a previous work [23]. In XRD results, it was clear that *β* is the predominant phase for both solution-treated and cold-rolled TNT alloy with a reduction ratio of 95%. *β*-phase appeared strong in both (110) and (211) planes. But for cold-rolled samples, more *α*″-peaks appeared stronger in (200) and (220) planes. This observed trend of the martensite phase increase is most probably due to the stress-induced martensitic transformation which occurs as a result of applying deformation load in such type of alloy (TNT) with the intermediate level of *β*-phase stability. This TNT alloy was designed to be located in the elastic instability phase zone of the bcc *β*-phase. Therefore, stress-induced martensitic transformation is expected in it and confirmed in this work.

With much heavier cold deformation reduction, it will lead to more martensitic-induced transformation and/or further change in the morphology of the martensite and the parent *β*-phases. Shown in Figure 2(c) is the microstructure of a wire with a reduction in the cross-sectional area of 90%. It was found that the martensite phase is very fine and only the deformation and slipping deformation bands are observed.

**Figure 3:**
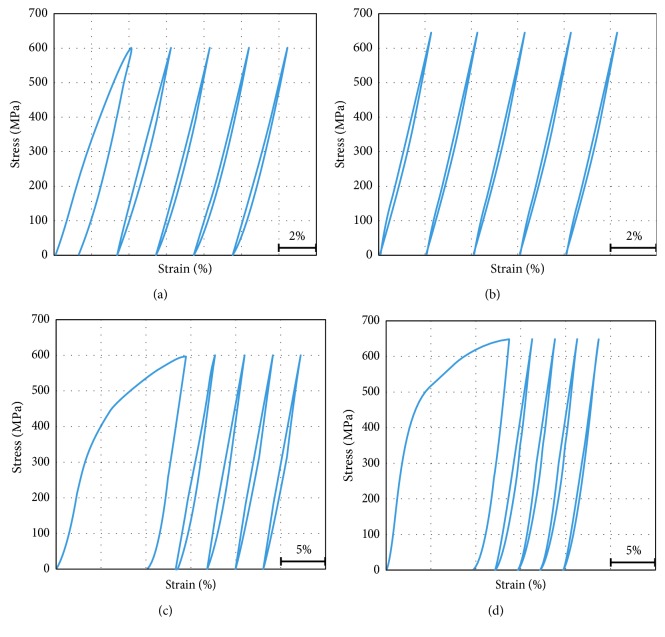
Cyclic tensile stress-strain curves of the CR plate up to (a) 600 MPa and (b) 650 MPa and ST plate up to (c) 600 MPa and (d) 650 MPa.

**Figure 4:**
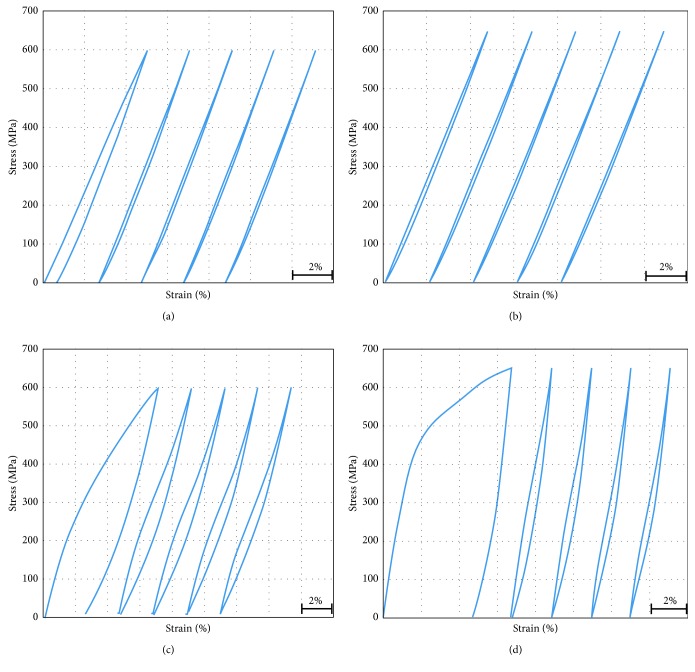
Cyclic tensile stress-strain curves of CR wire up to (a) 600 MPa and (b) 650 MPa and ST wire up to (c) 600 MPa and (d) 650 MPa.

Shown in Figures 3 and 4 are cyclic tensile stress-strain curves (up to selected ultimate stress limits 600 MPa and 650 MPa which are located in the stress level of martensite-induced stress range as extracted from its engineering stress-strain curves) of the TNT alloy specimens in the form of the plate and wire under ST and CR conditions.

The error in the measured strain values of the plate testing is minimum after the first cycle, where the slipping in the grip can be neglected after the first cycle. Also, the gauge length is considered 10 mm which is the total part of the parallel length. In spite of this, the strain error in the wire is high as there is no difference in the cross section in the wire along the whole specimen (inside the grip and along the gauge length).

General remarks on the cyclic loading and deloading tests as illustrated in Figures 3 and 4 showed that, except the first cycle, due to some apparent plastic deformation or/and slipping in the grips, all the samples return to almost their original size after deloading in all conditions under study. The hysteresis in the stress-strain cycle (loop) is a sign for the stress-induced martensitic transformation *β* ⟶ *α*″ during loading and its reverse transformation *α*″ ⟶ *β* during deloading. Also it was found that the hysteresis is higher in the ST that the CR condition in both plate and wire samples due to more *β* ⟶ *α*″ transformation. For some samples, it was clear that they restored more than 3.5% elastic strain, which is a high value of superelasticity in the present TNT alloy. And the *β* ⟶ *α*″ stress-induced transformation occurs along a wide range of stresses, up to 650 MPa. There is no significant difference between making ultimate stress to be 600 or 650 MPa as the sample returns to its original size after deloading regardless of the former loading stress. This gives us a room to have high elasticity (superelasticity) up to loading stress as high as 650 MPa.

**Figure 5:**
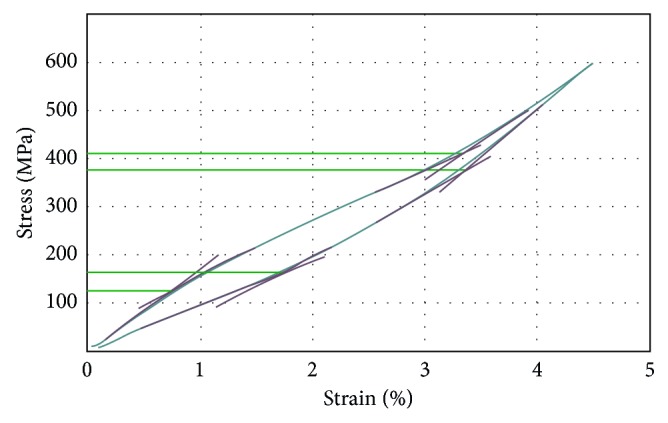
Typical stress-strain curve with superelastic evaluation showing the critical stresses measurements.

From the stress-strain cyclic loading and deloading tensile testing curves, the critical stress values of *σ*_Ms_, *σ*_Mf_, *σ*_As_, and *σ*_Af_ (defined as critical stresses at which the transformation *β* ⟶ *α*″ starts and finishes during loading and the reverse *α*″ ⟶ *β* transformation starts and finishes during deloading, respectively) were obtained from the fifth cycle for both flat and wire (ST and CR) specimens, and the critical stresses were calculated as illustrated in Figure 5.

**Figure 6:**
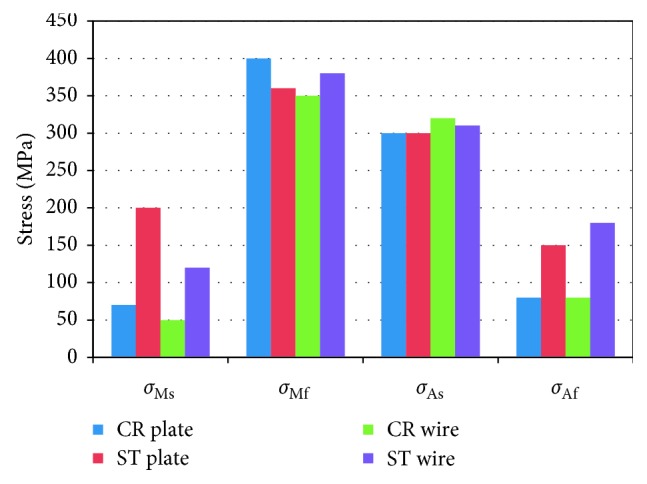
The critical transformation stress values of *σ*_Ms_, *σ*_Mf_, *σ*_As_, and *σ*_Af_ for flat and wire specimens in the ST and CR conditions.

The critical stress values of the alloy in the loading-deloading tests are measured and summarized in Figure 6 where the following observations were found: *σ*_Ms_ is lower in the case of CR specimens compared to ST specimens, and *σ*_Mf_ values range between 350 and 400 MPa. Also, it was found *σ*_As_ values are the same in the plates specimens which is 300 MPa, and in case of the wire sample, it exceeded this value with 10 to 20 MPa only. For CR specimens, *σ*_Af_ has lower values which are equal to 80 MPa, but ST specimens were 150 MPa and 180 MPa for plate and wire respectively. In general, the stress interval for the *β* ⟶ *α*″ transformation in loading is wider than that of the reverse *α*″ → *β* transformation in deloading. The stress interval for the *β* ⟶ *α*″ transformation in loading and the reverse *α*″ ⟶ *β* transformation in deloading of CR specimens are wider than that of ST specimens (both wire and plate).

**Figure 7:**
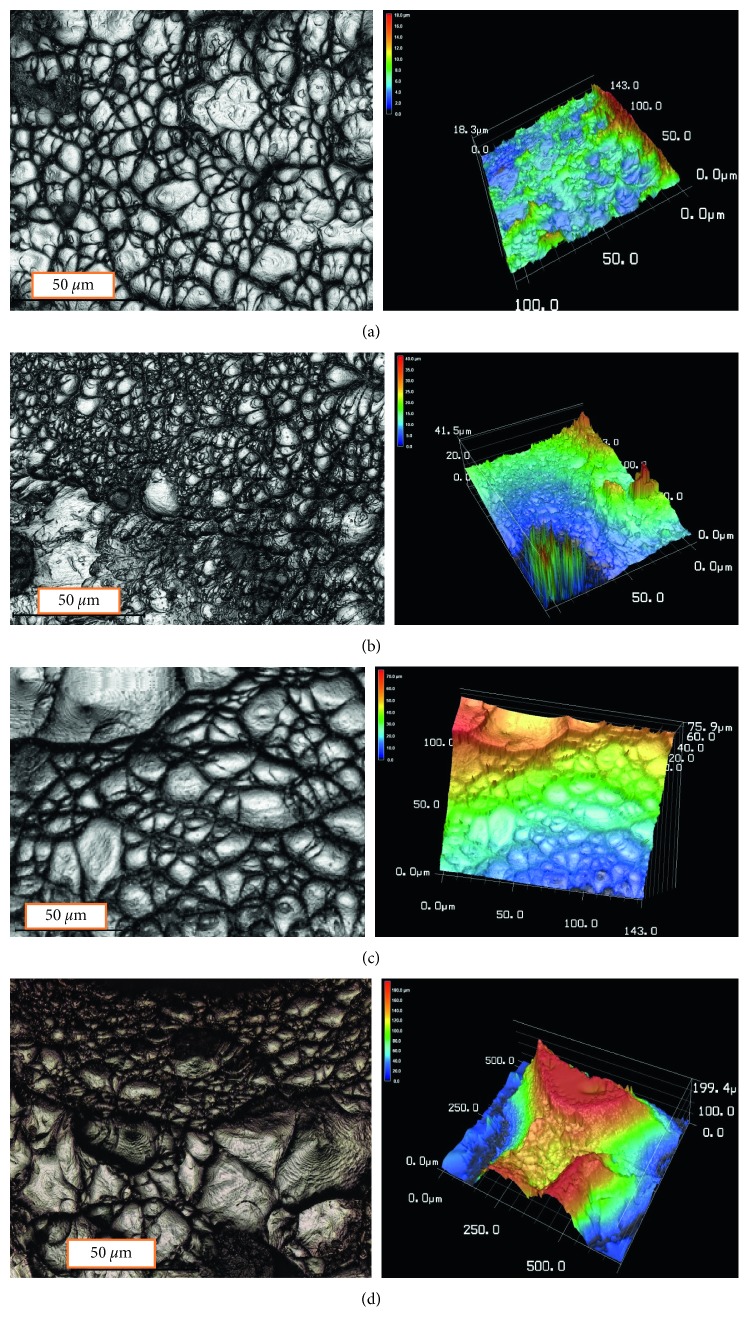
Fracture surface of the CR-ST plate and wire observed by the laser confocal microscope. The left ones are 2D optical images, while the right ones are 3D morphology laser images. (a) CR plate. (b) ST plate. (c) CR wire. (d) ST wire.

The graphs after failure of tensile tests samples for cold-rolled and solution-treated wire and plate specimens are illustrated in Figure 7. Investigating the fracture surfaces by the laser scanning microscope proved that the fracture was ductile. For all samples, dimples appeared in all fracture surfaces with different sizes. The dimples in solution-treated specimens are much smaller and shallower compared with the ones in cold-rolled specimens.

No cleavage was observed through the fractured figures, which is a good indicator for a perfect fracture plasticity of the specimens. In few, the ductile fracture mechanism was observed in all wire and plate conditions.

## 4. Conclusions

In this work, the superelasticity of the biocompatible Ti-17Nb-6Ta alloy was studied. Also, the microstructure and the modes of failure were investigated. It was observed that the *β*-phase is predominant in the ST condition and stress-induced *β* ⟶ *α*″ transformation is observed by cold rolling of the TNT alloy for both flat and wire conditions. The observed dimples in the fracture surface in the TNT alloy is an evidence of its good ductility. Also, dimples in the fracture surface of ST specimens are much smaller and shallower than in CR specimens. The *β* ⟶ *α*″ stress-induced transformation occurs along wide range of stresses in the alloy. In general, the stress interval for the *β* ⟶ *α*″ transformation in loading is wider than that of the reverse *α*″ ⟶ *β* transformation in deloading. In cyclic testing, after deloading, all the specimens returned to its original size after releasing the former loading stress (up to 650 MPa) with no significant difference between making ultimate stress to be 600 or 650 MPa. This gives a room to have high elasticity (superelasticity) up to loading stress as high as 650 MPa. TNT showed a recoverable elastic strain value up to 3.5%. For all samples, ST showed a wider hysteresis loops compared with CR loops which means that the microstructure affects the mechanical properties in the cyclic loading and deloading of the TNT alloy.

## Data Availability

The data used to support the findings of this study are available from the corresponding author upon request.
